# The prognostic role of RANK SNP rs34945627 in breast cancer patients with bone metastases

**DOI:** 10.18632/oncotarget.9356

**Published:** 2016-05-13

**Authors:** Arlindo Ferreira, Irina Alho, Inês Vendrell, Marta Melo, Raquel Brás, Ana Lúcia Costa, Ana Rita Sousa, André Mansinho, Catarina Abreu, Catarina Pulido, Daniela Macedo, Teresa Pacheco, Lurdes Correia, Luis Costa, Sandra Casimiro

**Affiliations:** ^1^ Instituto de Medicina Molecular, Faculdade de Medicina, Universidade de Lisboa, Lisbon, Portugal; ^2^ Oncology Division, Hospital de Santa Maria, Centro Hospitalar Lisboa Norte, Lisbon, Portugal; ^3^ Pathology Division, Hospital de Santa Maria, Centro Hospitalar Lisboa Norte, Lisbon, Portugal

**Keywords:** breast cancer, bone metastases, RANK/RANKL pathway, single nucleotide polymorphism, prognostic factor

## Abstract

Receptor activator of NF-kB (RANK) pathway regulates bone remodeling and is involved in breast cancer (BC) progression. Genetic polymorphisms affecting RANK-ligand (RANKL) and osteoprotegerin (OPG) have been previously associated with BC risk and bone metastasis (BM)-free survival, respectively. In this study we conducted a retrospective analysis of the association of five missense RANK SNPs with clinical characteristics and outcomes in BC patients with BM. SNP rs34945627 had an allelic frequency of 12.5% in BC patients, compared to 1.2% in the control group (*P* = 0.005). SNP rs34945627 was not associated with any clinicopathological characteristics, but patients presenting SNP rs34945627 had decreased disease-free survival (DFS) (log-rank *P* = 0.039, adjusted HR 2.29, 95% CI 1.04–5.08, *P* = 0.041), and overall survival (OS) (log-rank *P* = 0.019, adjusted HR 4.32, 95% CI 1.55–12.04, *P* = 0.005). No differences were observed regarding bone disease-free survival (log-rank *P* = 0.190, adjusted HR 1.68, 95% CI 0.78–3.66, *P* = 0.187), time to first skeletal-related event (log-rank *P* = 0.753, adjusted HR 1.28, 95% CI 1.42–3.84; *P* = 0.665), or time to bone progression (log-rank *P* = 0.618, adjusted HR 0.511, 95% CI 0.17–1.51; *P* = 0.233). Our analysis shows that RANK SNP rs34945627 has a high allelic frequency in patients with BC and BM, and is associated with decreased DFS and OS.

## INTRODUCTION

Receptor activator of NF-kB (RANK) and RANK-ligand (RANKL) are key regulators of bone remodeling by controlling the activity of osteoclasts [1, 2]. RANK and RANKL are also involved in bone tropism of breast cancer (BC) as well as the development of sex hormone-driven BC [3–5]. In human BC, RANK expression is mostly observed in hormone receptor-negative patients, being associated with higher proliferation index and histologic grade. It is also associated with a poor clinical outcome, including a higher incidence of bone metastases (BM) [6, 7]. Therefore, RANK-RANKL pathway inhibition is emerging as a clinically relevant therapeutic approach to prevent BC relapse, particularly bone relapse [8].

For that reason, it is important to investigate the biological and clinical effects of genetic variability within its major players. The pathologic relevance of genetic polymorphisms within RANK-RANKL pathway has been previously reported, in particular for conditions affecting bone physiology. Several studies have described single nucleotide polymorphisms (SNPs) in osteoprotegerin (OPG), RANK or RANKL, which influence bone mineral density (BMD), or are associated with susceptibility to rheumatoid arthritis, peri-implantitis, and sporadic primary hyperparathyroidism-related lower BMD [9–15].

Remarkably, two SNPs in OPG (rs3102735 and rs2073618), two SNPs in RANKL (rs9533156, rs1054016), and two SNPs in RANK (rs1805034 and rs35211496) have been shown to have a high allelic frequency in BC patients [16–18]. Amongst these only SNP rs1054016 in RANKL has been shown to have prognostic value, being associated with increased bone metastasis-free survival [18]. It was also shown that in patients with early stage, hormone-sensitive BC who were receiving therapy with aromatase inhibitors, the OPG SNP rs2073618 plus the RANKL SNP rs7984870 were associated with aromatase inhibitor-related musculoskeletal adverse events, a finding thought to be mediated by an increased RANKL/OPG ratio, higher levels of serum biomarkers of bone turnover, and a lower lumbar spine BMD [19].

In this study we investigated whether SNPs in RANK gene could be related to relevant clinicopathological characteristics and clinical outcomes in patients with BC and BM.

## RESULTS

### Characterization of RANK SNPs

**Figure 1:**
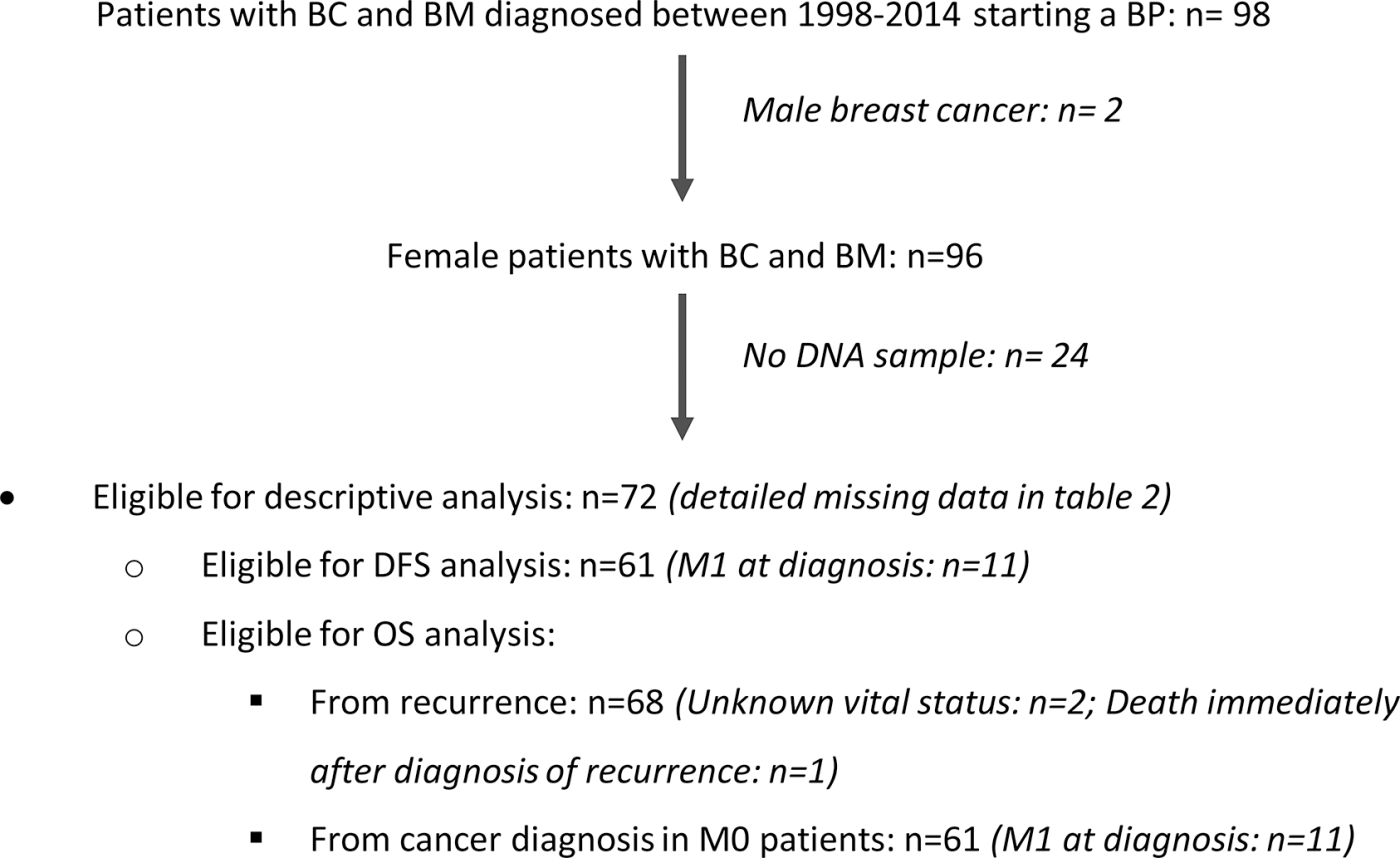
Patients' flowchart

Overall, 72 patients meeting eligibility criteria (Figure 1) and 80 age-matched healthy blood donors were available for SNPs evaluation. We studied five intragenic missense SNPs, which have known minor allelic frequencies below 5% in the general population (Table 1). SNP rs34945627 was found to have a disproportionally high allelic frequency of 12.5% (9/72) in BC patients, compared to 1.2% in the control group (1/80; *P* = 0.005) (Table 1). All patients with SNP rs34945627 were heterozygous. The remaining SNPs analyzed had an allelic frequency of 2.8% in BC patients, and only SNP rs12721431 was identified in two (2.5%) healthy women. RANK SNP rs34945627 induces an R450W alteration in the protein sequence that we hypothesize may impact the protein function. Therefore, we decided to further explore its association with clinical features and outcomes.

**Table 1:** SNP identification and characteristics

SNP	Mutation^a^	AA^b^	TaqMan Assay	Genotype	Breast cancer *n* (%)	Healthy *n* (%)	*P* value
rs34945627	1386C > T	R450W	C_25614163_10	CC CT	63 (87.5) 9 (12.5)	79(98.8) 1 (1.2)	**0.005**
rs12721431	888G > A	G284S	C_31393814_10	AA AG	70 (97.2) 2 (2.8)	78 (97.5) 2 (2.5)	0.915
rs35184120	1419G > A	V461M	C_25614164_10	GG GA	70 (97.2) 2 (2.8)	80 (100) 0 (0)	0.133
rs35993683	1435G > A	R466H	C_25614165_10	GG GA	70 (97.2) 2 (2.8)	80 (100) 0 (0)	0.133
rs61751992	1557G > A	A507T	C_90195803_10	GG GA	70 (97.2) 2 (2.8)	80 (100) 0 (0)	0.133

a Accession number NM_003839.2.

b Accession number NP_003830.1.

### Study sample

Patients' demographic and clinicopathological characteristics are presented in Table 2. On the whole cohort, median age at diagnosis of BC was 51.3 (interquartile range [IQR] 41.3–61.0) years. The majority of patients were metastatic at diagnosis (*n* = 61, 81.4%). Those not metastatic at diagnosis relapsed at distant sites after a median interval of 56 months (IQR 30.0–107.8), with bone-specific recurrence after a median interval of 76.2 months (30.6–114.3). The majority of tumors were hormone receptor-positive (*n* = 63, 90.0%) and HER2-negative (*n* = 43, 71.7%).

**Table 2:** Patients' demographics and clinical characteristics in the full cohort and according to RANK SNP rs34945627

	Full cohort	RANK SNP Genotypes		*P* value (*CC vs CT*)
		CC *n* (%)	CT *n* (%)	
Total, *n* (%)	72 (100)	63 (87.5)	9 (12.5)	–
Age (years) at diagnosis of breast cancer, median (IQR)	51.3 (41.3–61.0)	51.8 (41.1–60.4)	45.9 (43.5–62.8)	0.753
Age (years) at diagnosis of bone metastases, median (IQR)	58.0 (47.7–67.4)	58.0 (47.7–68.0)	59.2 (48.4–65.7)	0.953
Menopausal status, *n* (%)				0.592
Premenopausal	34 (47.2)	29 (46.0)	5 (55.6)	
Postmenopausal	38 (52.8)	34 (54.0)	4 (44.4)	
Metastatic at diagnosis, *n* (%)				1.000
M0	61 (81.4)	53 (84.1)	8 (88.9)	
M1	11 (18.6)	10 (15.9)	1 (11.1)	
Hormone receptor status, *n* (%)				1.000
ER and/or PR positive	63 (90.0)	55 (90.2)	8 (88.9)	
ER and PR negative	7 (10.0)	6 (9.8)	1 (11.1)	
Unknown	2 (2.8)	2 (3.2)	0 (0)	
HER2 receptor status, *n* (%)				0.676
Positive	17 (28.3)	14 (26.9)	3 (37.5)	
Negative	43 (71.7)	38 (73.1)	5 (62.6)	
Unknown	12 (16.7)	11 (17.5)	1 (11.1)	
Radiographic pattern of bone metastasis, *n* (%)				0.203
Lytic	40 (61.5)	35 (60.3)	5 (71.4)	
Blastic	10 (15.4)	8 (13.8)	2 (28.6)	
Mixed	15 (23.1)	15 (25.9)	0	
Unknown	7 (9.7)	5 (7.9)	2 (22.2)	
Extra-bone metastasis, *n* (%)				0.420
Yes	22 (31.4)	41 (66.1)	7 (87.5)	
No	48 (68.6)	21 (33.9)	1 (12.5)	
Unknown	2 (2.8)	1 (1.6)	1 (11.1)	
NTX at diagnosis of bone metastases (nM BCE), *n* (%)				0.554
< 50	12 (18.5)	11 (19.3)	1 (12.5)	
50–100	13 (20.0)	10 (17.5)	3 (37.5)	
> 100	40 (61.5)	36 (63.2)	4 (50.0)	
Unknown	7 (9.7)	6 (9.5)	1 (11.1)	

ER – Estrogen receptor, IQR – interquartile range, PR – progesterone receptor, NTX – N-terminal collagen telopeptide, BCE – Bone collagen equivalents.

### Association of RANK SNP rs34945627 with clinical features and outcomes

We subsequently investigated if SNP rs34945627 was associated with relevant clinicopathological characteristics in patients with BC and BM. As detailed in Table 2, SNP rs34945627 does not seem to be associated with any of the selected characteristics.

To assess a putative prognostic role of SNP rs34945627, we further tested its association with relevant disease outcomes, such as disease-free survival (DFS) and overall survival (OS), and bone-specific outcomes, such as bone disease-free survival (bDFS), time to first skeletal-related event (TTSRE), and time to bone progression (TTBP).

Median follow-up for DFS analysis was approximately 4.5 years (56.3 months, IQR 30.0–107.8), while median OS follow-up was approximately 4 years (48.2 months, IQR 27.0–82.2). During this period all non-metastatic patients at diagnosis recurred, as per study design, and 45 patients died: 36 (61%) in the SNP rs34945627 negative group and 9 (100%) in the SNP rs34945627 positive group. Date of disease recurrence was balanced between groups (*P* = 0.225).

**Figure 2:**
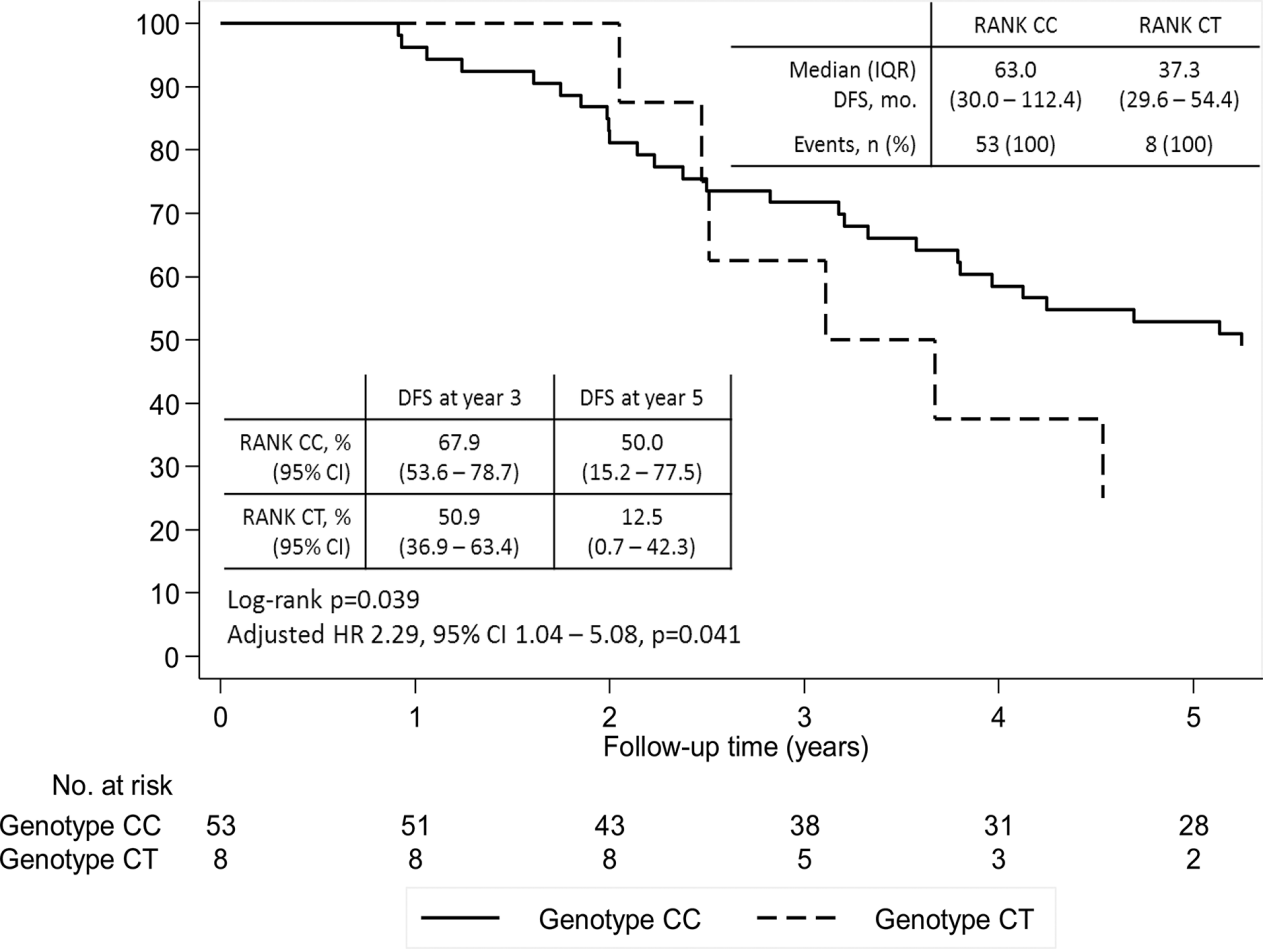
Disease-free survival (DFS) according to SNP rs34945627 Adjusted hazard ratio controlling for age at diagnosis.

When restricting to patients not metastatic at diagnosis, DFS was shorter in the group of patients heterozygous for SNP rs34945627, both in the univariate and multivariate analysis controlling for age at diagnosis (adjusted-hazard ratio (HR) 2.29, 95% CI 1.04–5.08, *P* = 0.041) (Figure 2). This effect reflects mostly a difference between groups after two years of follow-up, with a DFS at year five of 50% (95% CI 15.2–77.5) for wild-type patients versus 12.5 % (95% CI 0.7–42.3) for heterozygous patients.

**Figure 3:**
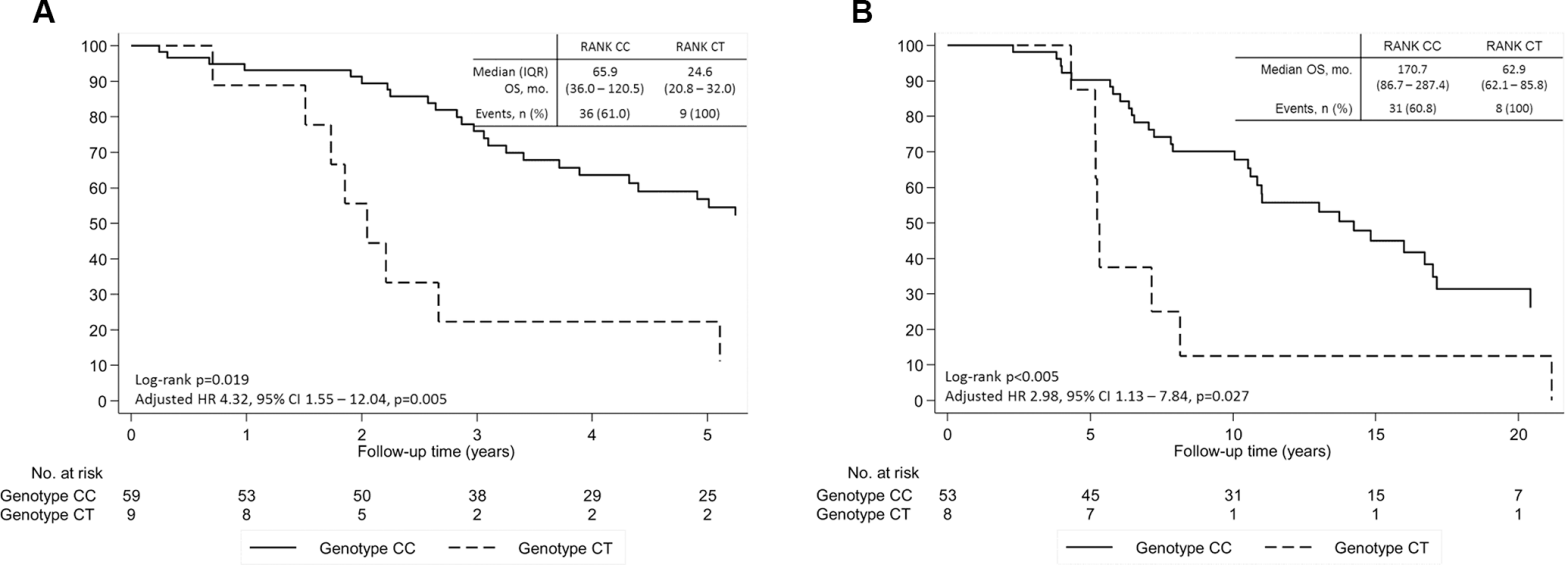
Overall survival (OS) of patients with breast cancer and bone metastases according to SNP rs34945627 (**A**) From time of diagnosis of metastatic disease. (**B**) From time of breast cancer diagnosis in patients cM0 at breast cancer diagnosis. Adjusted hazard ratio controlling for age at diagnosis, extra-bone metastases and NTX at diagnosis of bone metastases.

**Figure 4:**
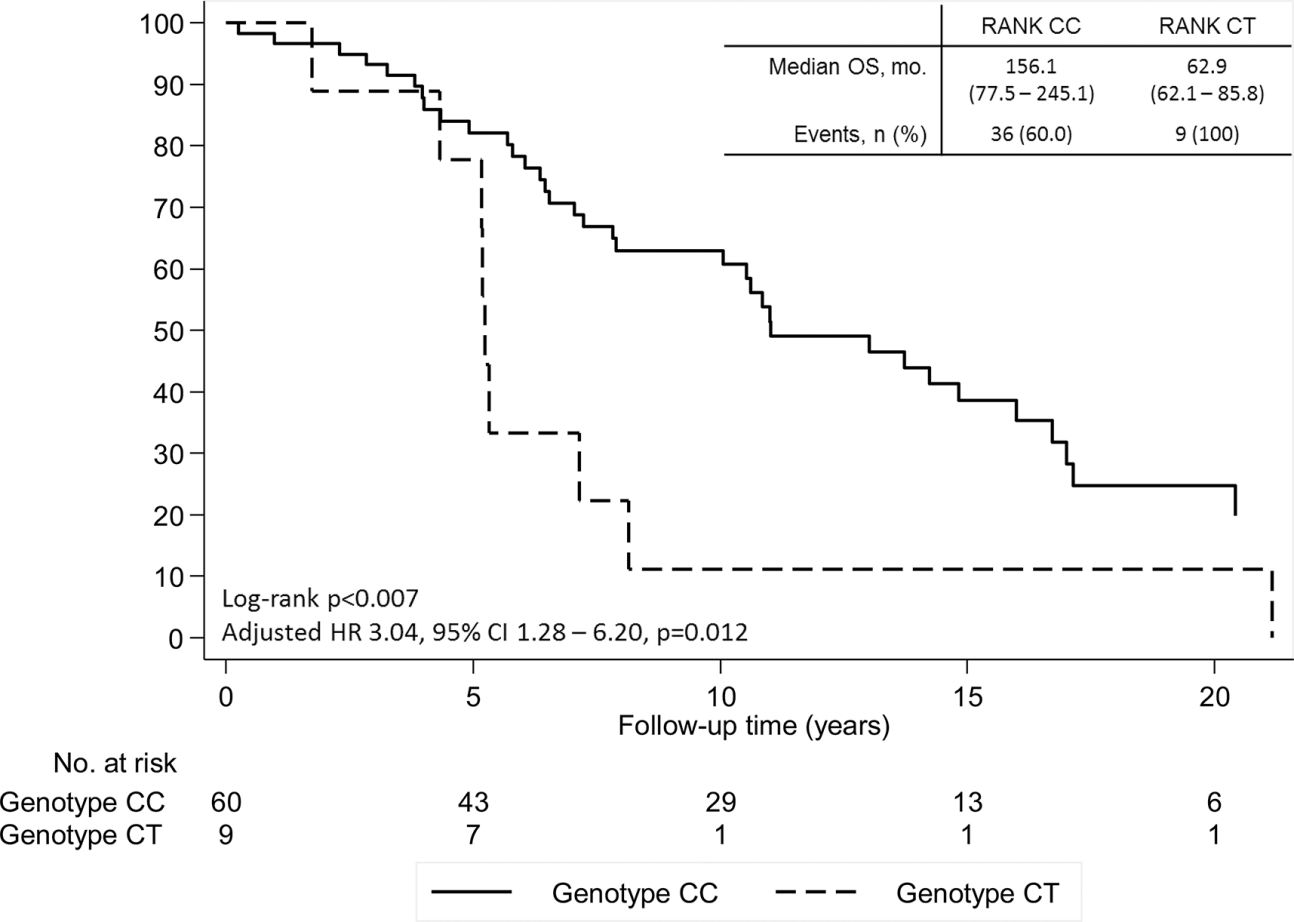
Overall survival (OS) of patients with breast cancer and bone metastases according to SNP rs34945627 from time of breast cancer diagnosis in the overall cohort Adjusted hazard ratio controlling for age at diagnosis, extra-bone metastases and NTX at diagnosis of bone metastases.

Patients presenting SNP rs34945627 also presented a decreased OS both in the univariate and multivariate analysis controlling for age at diagnosis, extra-bone metastases and NTX at diagnosis of BM (adjusted HR 4.32, 95% CI 1.55–12.04, *P* = 0.005; Figure 3A). This association was also present when analyzing OS from date of diagnosis of primary BC in cM0 patients (adjusted HR 2.98, 95% CI 1.13–7.84, *P* = 0.027) (Figure 3B) and in the overall cohort (adjusted HR 3.04, 95% CI 1.28–6.20, *P* = 0.012) (Figure 4).

**Figure 5:**
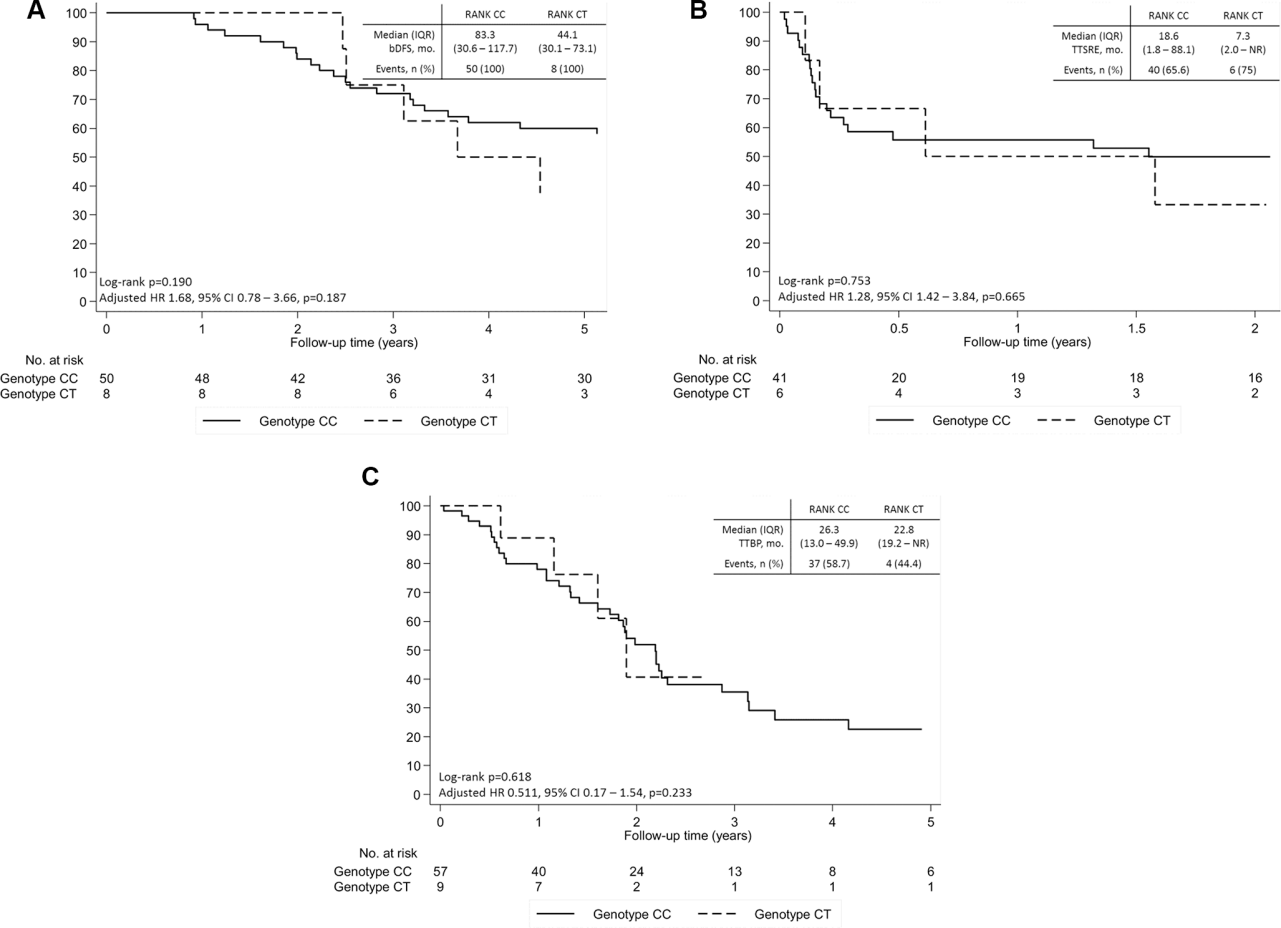
Bone disease-related outcomes of patients with breast cancer and bone metastases according to SNP rs34945627 (**A**) Bone disease-free survival. (**B**) Time to first skeletal-related event (SRE). (**C**) Time to bone progression. Adjusted hazard ratio controlling for age at diagnosis, radiographic pattern of bone metastases and NTX at diagnosis of bone metastases.

**Figure 6:**
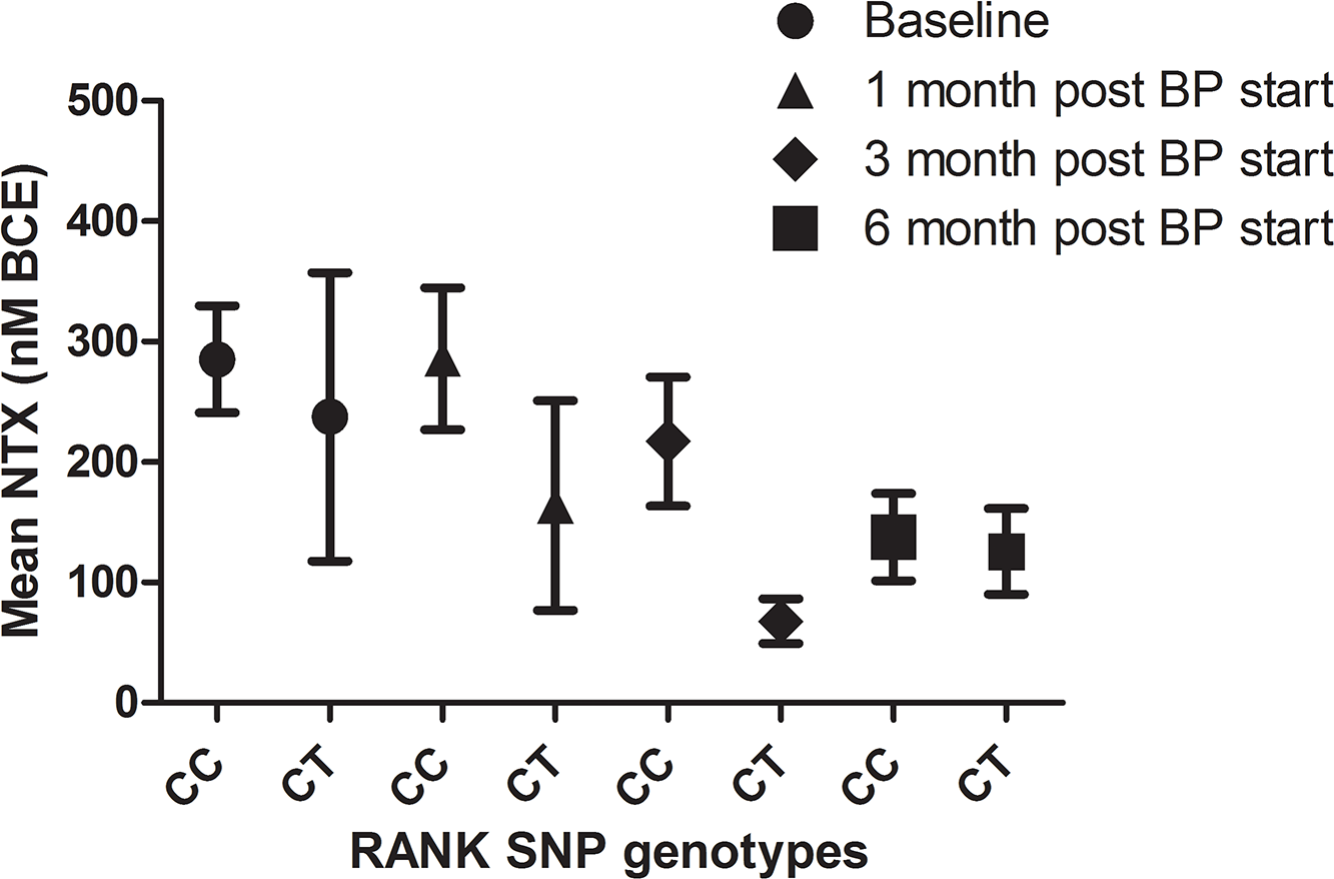
NTX values of patients with breast cancer and bone metastases according to SNP rs34945627 after bisphosphonates treatment (BPs) Results are expressed as the mean ± SEM.

No differences were observed in regard to bDFS (adjusted HR 1.68, 95% CI 0.78–3.66, *P* = 0.187), TTSRE (adjusted HR 1.28, 95% CI 1.42–3.84; *P* = 0.665), or TTBP (adjusted HR 0.511, 95% CI 0.17–1.51; *P* = 0.233), controlling for age at diagnosis, radiographic pattern of BM and NTX at diagnosis of BM (Figure 5). Moreover, no significant differences were observed between groups in NTX values during treatment with bisphosphonates (BPs) (Figure 6).

## DISCUSSION

In this study we show that from five selected RANK missense SNPs, SNP rs34945627 is more common in women with metastatic BC when compared to healthy women. Furthermore, patients presenting SNP rs34945627 did not show any particular clinical or pathological feature, namely regarding bone disease, but had a worse prognosis in terms of DFS and OS.

RANK-RANKL pathway is a key regulator of bone physiology [1, 2], but also a central player in the onset of BM, as RANKL acts as a chemoattractant for RANK-positive tumor cells [20, 21]. Other studies also revealed its role in tumor cell tropism for sites outside bone, such as lung [22].

Besides its role in bone, RANK-RANKL signaling pathway has also a key role in mammary gland development [23, 24]. Importantly, it was demonstrated that progesterone-induced RANKL signaling in luminal mammary epithelial cells not only activates RANK downstream pathway in an autocrine way but also activates RANK signaling in the RANKL-negative basal mammary epithelial cells in a paracrine manner [24]. In these cells, the downstream activation of the IKKa-NFkB-Cyclin D1 axis results in proliferation and expansion of mammary stem cells. Therefore, it was reasonable to postulate that RANK-RANKL axis could be involved in mammary gland tumorigenesis and it was further demonstrated that RANK-RANKL signaling induces hormone-dependent BC [4].

In this study we found that BC patients presenting SNP rs34945627 had a worse prognosis in terms of DFS and OS. Based on our results we hypothesize that the genetic alteration of RANK induced by the SNP rs34945627 may disturb the RANK-RANKL pathway in the tumor cells and/or in the host RANK-expressing cells, like osteoclasts. RANK pathway activation upon RANKL binding to the receptor involves the recruitment of tumor necrosis factor receptor-associated factors (TRAFs), and TRAF2 and 6 are required for the JNK, p38, ERK, PI3K/mTOR, NFƙB downstream activation [25, 26]. These pathways are responsible for RANK-RANKL effect over cell proliferation, survival, growth, and osteoclasts differentiation. RANK SNP rs34945627 induces an R450W mutation that we hypothesize may affect TRAF2 and/or TRAF6 recruitment since the affected residue, amino acid 450, has been identified as part of TRAF2 and 6 binding sites [25, 26]. This would compromise downstream effectors activation, thus leading to altered cell phenotypes. Therefore, RANK-positive tumors harboring this mutation may be more aggressive by exhibiting exacerbated RANK-RANKL pathway-related phenotypes, like proliferation, epithelial-to-mesenchymal transition, invasiveness and stemness [6, 21]. This could explain the effect observed in DFS. In contrast, other SNPs in RANK gene that were shown to have high allelic frequencies in patients with BC, namely rs1805034 and rs35211496 (minor allelic frequency of 47.8% and 17.3%, respectively), had no prognostic value [18].

Additionally, osteoclasts with R450W mutation could have a RANKL-independent activation of RANK, leading to a more favorable bone niche for BC relapse. However, bDFS, TTSRE and TTBP were equal amongst groups favoring the hypothesis that the effect will be tumor cell-related. Moreover, the fact that the patients included in this study were all treated with BPs, may explain why we do not observe any differences in terms of TTSRE and TTBP.

This study results raise important questions that we are currently addressing in the laboratory. Will the R450W mutation result in gain or loss-of-function of RANK? Does it stimulate tumor growth or ability to invade and metastasize? Does it distress osteoclasts and how? What is the impact on response to RANKL-targeted therapies?

BC therapy has evolved significantly over the last three decades with the universal access to adjuvant treatments and its incremental improvement over time [27–29]. Identifying patients at higher risk of death, especially in the sub-group of hormone-receptor positive tumors, has been an area of intense research with the development of molecular genetic signatures and other risk scores [30, 31]. Disease risk stratification enables the adjustment of therapy intensity, which is now a standard medical practice and a relevant component in improving BC care outcomes [32]. In this area, the validation of RANK SNP rs34945627 as a prognostic marker for high risk of relapse and death in BC patients would contribute to this endeavor of adjusting the most appropriate adjuvant therapy intensity.

Although RANK-RANKL pathway inhibition is emerging as a clinically relevant therapeutic approach to prevent BC relapse, prognostic and predictive factors of the need and benefit from RANKL blockage are still missing. In this study, we show that BC patients with BM carrying a specific SNP in the RANK gene have a significantly worse prognosis, as measured by shorter DFS and OS, thus identifying a subset of patients in need for optimized therapies. Moreover, we show that in this cohort that includes only patients from the pre-denosumab era there are no differences concerning bone-related outcomes. Therefore, understanding how this RANK polymorphism is related with response to denosumab is an intriguing point to explore in future studies.

In conclusion, our study shows that RANK SNP rs34945627 is more common in BC patients than in age-matched healthy controls. Although patients presenting RANK SNP rs34945627 do not seem to differ in terms of demographic and tumor clinicopathological characteristics, or bone disease onset and evolution, these patients do worse in terms of DFS and OS. These findings require further studies to address their implication in the clinical management of BC.

## MATERIALS AND METHODS

### Study population and design

In this retrospective cohort study we included patients followed at Oncology division from Santa Maria Hospital, Lisbon, Portugal, diagnosed with BM from BC between 1998 and 2014, and starting therapy with bisphosphonates (BPs). A total of 72 patients, with available peripheral blood collected at the time of first treatment with BPs, were included.

Cancer treatment was provided as per institutional guidelines, in compliance with international guidelines at the time of diagnosis. We then retrospectively collected a set of demographic and clinicopathological information, namely: age at diagnosis of primary BC and BM, menopausal status, metastatic disease at BC presentation, hormone receptors and HER2 status in primary BC, date and site of disease recurrence and progression, radiographic pattern of BM, number and timing of SREs, urinary NTX at diagnosis of BM (cut off value of 100 nmol BCE/mmol creatinine) and survival.

A control group of 80 female healthy blood donors was selected from the Biobanco-IMM (http://biobanco-imm.biobanco.pt/), based on median age at diagnosis of primary BC in the patients' group.

This study was ethically approved by local Institutional Review Board, and complies with all national regulations.

### SNP selection and genotyping

Five known SNPs within the RANK gene locus were selected based on the following parameters: 1) intragenic exonic location; 2) causing missense alterations; 3) low allelic frequency (HapMap or 1000Genomes datasets with a minor allele frequency ≤ 5%); 4) not previously investigated in cancer-related studies (Table 1).

DNA was extracted from whole blood using column-based commercial methods, according to manufacturer's instructions. DNA was quantified by spectrophotometry in a NanoDrop, and stored at −20°C. Alleles were genotyped using pre-designed TaqMan assays, in an Applied Biosystems 7500 Fast Real-Time PCR System. Results were analyzed using the TaqMan Genotyper Software.

### Statistical analysis

Demographic and clinicopathological characteristics of the full cohort are described using frequencies for categorical variables and central tendency, dispersion and range for continuous variables. Univariate association of these characteristics and SNPs was performed using Fisher's exact test and Wilcoxon rank-sum test. Survival and cumulative incidence plots were performed using Kaplan–Meier methods. Univariate differences between survival rates were tested for significance using the log-rank test; while multivariate analysis for survival was tested using Cox proportional hazards models. Disease-free survival (DFS) and bone disease-free survival (bDFS) were defined as time from BC diagnosis to date of recurrence or death of any cause, or date of bone-specific recurrence or death of any cause, respectively. Overall survival (OS), if not otherwise specified, was defined as time from disease recurrence to death of any cause. Time to first skeletal-related event (TTSRE) and time to bone progression (TTBP) were defined as time from bone-disease recurrence to first SRE or bone-disease progression, respectively. All patients with missing data in relevant variables were excluded from the multivariate analysis. Analyses were performed using Stata 13.1 software (StataCorp LP).
